# A Case of Laryngotomy for the Cure of Roaring

**Published:** 1889-04

**Authors:** Daniel B. Lee

**Affiliations:** Instructor in Anatomy and Operative Veterinary Surgery, Harvard University


### ART. XI.—A CASE OF LARYNGOTOMY FOR THE CURE OF ROARING.

BY DANIEL B. LEE, M. D. V.,

*Instructor in Anatomy and Operative Veterinary Surgery, Harvard University.*

After reading the numerous articles on the subject of laryngotomy and excision of the left vocal cord which appeared in the veterinary publications last summer, I became much interested in the subject, and after having tried the operation on a dog, kid and heifer, as well as on a dead horse, I was finally enabled to operate on a very bad case of roaring which occurred in the practice of Dr. Alderman of East Lexington.

The patient was a very coarse built farm horse, who had been previously treated for influenza, enteritis, and laminitis, which followed one another, soon after the horse was bought. The horse had *never* been known to make any vocal sound, but when driven at a slow trot for a short distance he began to roar very badly.

Having decided to operate, we cast the horse and turned him on his back. I then laid open the larynx and found that the *vocal cords were absent*, this accounting for the fact that the horse had never made any vocal sound. The mucous membrane of the larynx was thickened and showed several processes of cartilaginous nature which were situated where the attachments of the vocal cords should have been. These I cut out and some hemorrhage resulted, which was soon controlled, and the wound being left open, the horse was allowed to rise. As soon as he regained his feet he was seized with a terrible attack of dyspnœa, which must have been caused by spasm of the epiglottis brought on by the irritation of the mucous membrane of the larynx from the operation, for very little blood ran down the trachea to the lungs, and the dyspnœa was at once relieved by tracheotomy, which would not have been the case if blood had entered the bronchial tubes. At the end of half an hour I took out the tracheotomy tube without causing the least inconvenience, but when a pail of water was offered to the horse the dyspnœa at once returned when he attempted to swallow. The tube was again introduced into the trachea and relief was complete. For the next two weeks the tube was only used at night, the symptoms of dyspnœa never returning.

One remarkable symptom was noticed, namely, that when water was taken a small amount escaped by both the opening in the larynx and that in the trachea. In the third week the tracheotomy tube was permanently removed and the opening in the trachea allowed to close. At the end of six weeks both wounds were healed, and the horse when driven at a fast trot could not be made to roar. It is now nearly five months since the operation and same is complete. The cause of roaring was evidently the presence of the cartilaginous processes in the larynx, these being caused by some ulcerative process which brought about the destruction of the vocal cords, their attachments only remaining, and the horse at the same time being rendered dumb.

In the case of the heifer which I operated on as an experiment, I noticed that when water was taken it escaped in small quantities from the opening in the larynx ; I also noticed that when she chewed her cud there was a very slight discharge, most noticeable after eating grass, when it was green in color. In both the horse and the heifer these symptoms only lasted four or five days, and can be accounted for very easily in that there was of course a great deal of swelling of laryngeal mucous membrane and surrounding tissues, and this naturally made the action of the epiglottis in closing the opening of the larynx less perfect than round.

I should like to call the attention of the profession to the fact that if the operation of excision of one or both vocal cords was performed on milk-cows that are kept in thickly settled towns, the animals being thus rendered dumb could not annoy the neighborhood by their bellowing when in heat or after their calves are taken from them.

I have never found that the fact of being dumb troubles the animal in the least, the heifer, which I still have, goes through all the motions necessary to produce sound, but failing to do so appears perfectly satisfied. Of course, cattle running at pasture could not be so treated, as they are more exposed to accidents and need their vocal power to call assistance, but for those kept in stables near dwellings houses, I see no drawback, and certainly a source of great annoyance to people living near would be removed.

