# Notes—Errata

**Published:** 1889-07

**Authors:** 


NOTES—ERRATA.—In Dr. D. D. Lee's article on Laryngotomy April No., page 161, last word of second paragraph the word "round" should be "normal," so as to read "the opening of the larynx less perfect than normal."

CONVENTION FOR THE REVISION AND PUBLICATION OF THE PHARMACOPŒIA OF THE UNITED STATES, will be held in Washington May 7, 1890. All medical colleges and associations desiring to be represented will send their corporate title and list of officers to Dr. Edwin H. Brigham, 19 Boylston Place, Boston.

ELEVENTH CENSUS.—Dr. John S. Billings, Surgeon U. S. Army, is to take charge of the Report on the Mortality and Vital Statistics.

The census office will issue to the medical profession throughout the country "Physician's Registers" for the purpose of obtaining more accurate returns of death than it is possible for the enumerators to make. Physicians not receiving registers can obtain them by sending their names and addresses to the census office. It is earnestly hoped that physicians in every part of the country will co-operate with the census office in this important work.

